# Taylor's approach in an ankylosing spondylitis patient posted for percutaneous nephrolithotomy: A challenge for anesthesiologists

**DOI:** 10.4103/1658-354X.57879

**Published:** 2009

**Authors:** Parul Jindal, Gaurav Chopra, Amit Chaudhary, Aslam Aziz Rizvi, J. P. Sharma

**Affiliations:** *Department of Anesthesiology, Intensive Care and Pain Management, Himalayan Institute of Medical Sciences, Dehradun, Uttaranchal, India*

**Keywords:** *Ankylosing spondylitis*, *Taylor's approach*, *percutaneous nephrolithotomy*

## Abstract

We describe a patient with long-standing ankylosing spondylitis who underwent percutaneous nephrolithotomy under spinal anesthesia. At preoperative assessment, it was considered that intubation of the trachea was likely to be difficult. Fiberoptic bronchoscopy was attempted, but without success. As the standard technique for spinal anesthesia failed, a variation of the paramedian approach in the lumbosacral approach, also known as Taylor's approach was successfully attempted. This resulted in adequate sensory and motor blockade for the surgical procedure. The patient did not require airway interventions, but equipment and aids to secure airway were available.

## INTRODUCTION

Ankylosing spondylitis (AS) is a chronic inflammatory disease of the joints. Its main characteristic is the fusion of the bones in the spine, which causes loss of flexibility of the back and neck. It usually begins between the second and the fourth decades of life, mainly affecting males (5:1) and HLA-B27 positive[1] individuals. AS patients present specific challenges to the anesthetist. Both airway management and neuraxial access may prove to be difficult. The trend has been to deal with the airway challenge, and avoid neuraxial anesthesia.[2]

## CASE REPORT

A 52-year-old male, weighing 54 kg, height 164 cm, with a 22-year-old history of ankylosing spondylitis was admitted in the Urology Department, for percutaneous nephrolithotomy.

**Figure 1:**
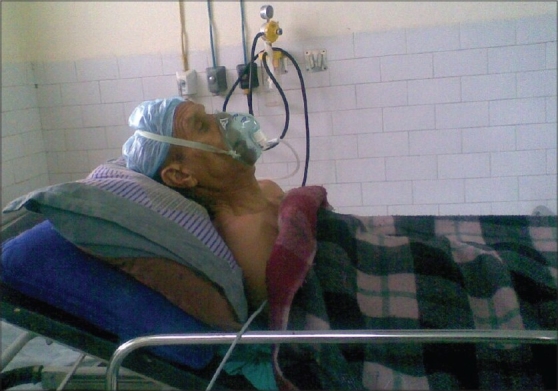
Patient in the recovery room. Due to the ankylosing spine, the patient's head is supported with two pillows and the head end of the bed is raised

During preanesthetic assessment his history revealed that the he required three pillows to support his head due to the disease process and involvement of the cervical spines [Figure 1]. A physical examination suggested that there was severe cervical spondylitis involving thoracolumbar vertebral column, without lower limb neurological involvement. No cardiovascular system abnormality was detected.

The airway examination showed adequate mouth opening with artificial dentures, Mallampati grade III restricted neck mobility. A preoperative assessment for difficult intubation was made.

Radiographs of the vertebral column revealed ankylosis of the cervical, thoracic, and lumbar spinous processes showing posterior joint involvement, resorption of the anterior surfaces of the vertebral bodies and calcification and ossification of the posterior ligaments and surrounding soft tissues.

Following the traditional anesthetic approach to ankylosing patients, we decided to opt for securing the airway using awake endotracheal intubation; however, the patient did not give his consent for this. A difficult intubation cart, which had a selection of oropharyngeal, nasopharyngeal airway, gum elastic bougie, fiberoptic bronchoscope, cricothyroidotomy, and other aids, was kept ready.

On the day of the surgery, the patient was premedicated with oral ranitidine 150 mg and diazepam 5 mg, two hours before surgery, with a sip of water, and injection glycopyrrolate 0.2 mg intramuscularly one hour before surgery. Preparation for fiberoptic intubation was done. Nasal patency was checked and the nasal cavity prepared with oxymetazoline nasal drops. In the operation room the patient was made to lie supine with the head adequately supported on three pillows and routine monitors, such as, the electrocardiogram, non-invasive blood pressure, and pulse oximeter and capnograph were placed. The IV line, with a 18 Gauge canula, was started. Anesthesia was induced with IV injection propofol 2 mg/kg, slowly, in titrated doses, with fentanyl 2 μg/kg. The anesthetist had to use a higher footstep than normal to get the desired level. We opted for fiberoptic bronchoscopy, but as the heart rate increased to 144/min and the blood pressure increased to 180/118 mmHg, we administered a further bolus dose of 50 mg of propofol to increase the depth of anesthesia. The patient's heart rate, and blood pressure stabilized, but the patient started desaturating with apneic spells, so we decided to abandon the procedure. The patient was allowed to wake up. Regional anesthesia was taken as the next option. Even after three attempts by expert hands no cerebrospinal fluid (CSF) was obtained through the spinal needle.

Taylor's approach was attempted. Clear CSF was obtained through the spinal needle and 3 ml of 0.5% bupivacaine and 25μg/kg fentanyl was injected intrathecally. Due to the spinal deformity, we were not able to achieve the appropriate position for intrathecal block. Therefore, we carried out some maneuvres; we placed a pillow beneath the left shoulder to make the spine of the scapula perpendicular to the table, and the operating table (OT) was tilted 20° to the right, to straighten the spine and to make it parallel to the floor.

The block was adequate with sensory block up to T4 and adequate motor blockade upto T6. The vital signs remained stable. Fluid calculations and management of blood pressure were very important in this patient with severe lung disease. Prone position had to be given carefully, with bolsters confirming to the contours of the patient, to avoid fractures.

There were no intraoperative problems throughout the procedure, which was completed within three hours. Hemostasis was adequate. Supplementary oxygen was administered continuously during the surgical procedure. There were no postoperative sequelae to the spinal technique.

## DISCUSSION

Difficult airway is a challenge to anesthesiologists. Reduced range of motion or fixed cervical spine in patients of Ankylosing Spondylitis is a major problem in anesthesia.[2–4] Such patients are usually managed along the awake limb of difficult airway algorithm.

The anesthesiologist should base the anesthetic conduct on the extension of the disease, centered in four main aspects: Degree of upper airways involvement, presence of pulmonary restriction, degree of cardiac involvement, and access to the neuroaxis.[5]

The criteria that predict difficult airways should be reviewed, such as, the Mallampati test, Wilson index, thyromental distance, sternomental distance, the degree of head and neck movements, and mouth opening.[6]

Patients in the initial stages or with nonprogressive disease may have good neck mobility. Cervical involvement, with limited movements and in flexion, hinders tracheal intubation. A problem of intubation with a standard laryngoscope in such cases is due to nonalignment of the oral/pharyngeal and laryngeal axes making intubation difficult. One should avoid forcing the neck, even in the presence of neuromuscular blockade, due to the risk of fractures and the possibility of vertebrobasilar insufficiency, if possible cervical support can be used.[7]

Cardiological evaluation (electrocardiogram and echocardiogram) is essential to determine the cardiovascular risk. Involvement of the heart valves, especially the aortic valve, may be present, with associated conduction defects. The sudden and intense variation in systemic vascular resistance caused by the spinal anesthesia is not tolerated by patients with defects in the aortic valve. External cardiac massage in the presence of a rigid thoracic wall may be ineffective.[3]

A chest X-ray can show restrictive changes and, in a majority of the cases, pulmonary function tests should be requested. Respiratory insufficiency and the limitation of chest expansion increase the incidence of pulmonary complications and the need for postoperative mechanical ventilation in the ICU, especially in major surgeries.[8] X-ray of the lumbar spine may be useful to evaluate the possibility of spinal anesthesia.

The modified New York criteria, which combine clinical and radiological data, are used to confirm the diagnosis of AS. The clinical criteria are: Lumbar pain lasting for more than three months, which improves with exercise and does not improve with rest, limitation of the movements of the lumbar spine in the frontal and sagittal planes, and decreased thoracic expansibility. The radiological criteria are: Grade 2, 3, or 4 bilateral sacroiliitis and grade 3 or 4 unilateral sacroiliitis. The presence of one clinical and one radiological criterion is necessary for the diagnosis.[4] The involvement of the temporomandibular joint limits mouth opening in up to 40% of the patients, which can evolve to complete ankylosis.[7] Although rare, cricoarytenoid arthritis with dyspnea, stridor, and fixation of the vocal cords may be present.[8]

Fibroscope-guided intubation with mild sedation of the patient and anesthesia of the mucous membranes is the method of choice in patients with advanced deformity of the cervical spine.[10]

Neuroaxis blocks are technically difficult, due to limited articular mobility and obliteration of the interspinal spaces and due to the impossibility to position the patient adequately. Technical difficulties can also increase the risk of complications.[11] Care is required during administration of spinal anesthesia, because excessively high levels of block, with respiratory insufficiency, have been reported. Cases of spinal cord hematomas after epidural anesthesia have been reported.[12,13]

**Figure 2:**
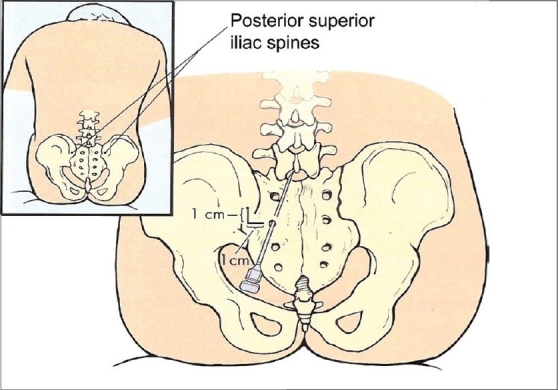
Diagrammatic presentation of Taylor's approach (reproduced from Batra M, Mulroy M, Neal J. Atlas of Anesthesia: Principles of Anesthetic Techniques and Anesthetic Emergencies)

In this case spinal anesthesia was considered, after unsuccessful attempts to establish the airway. As we failed to perform a subarachnoid block by the standard midline approach, due to anatomical/technical difficulties, we attempted the Taylor's approach [Figure 2], which is a modification of the paramedian approach for spinal anesthesia. It has all the advantages of the paramedian approach and also it is carried out at the L5-S1 interspace, the largest interlaminar space of the vertebral column. A spinal needle is inserted in a cephalomedial direction through a skin wheal raised 1 cm medial and 1 cm caudad to the lowermost prominence of the posterosuperior iliac spine.[14,15] We succeeded by this approach in the first attempt.

The spread of local anesthetics in the subarachnoid space depends on:

1. Gravity
2. Volume
3. Mass
4. Baricity
5. Speed of injection
6. Size of needle

Hyperbaric solutions (SG > 1.009) will tend to spread caudally in patients in the sitting position and in a cephalad direction in patients in the Trendelenberg position. On account of the intrinsic curves of the spine, an injection made at L4 or lower, with the patient in the supine position, will tend to spread caudally and that made at L3 or higher will spread in the cephalad direction. Hypobaric solutions (SG < 1.003) will spread in the opposite direction.[16,17]

In our case we achieved two goals, that is, alignment of the vertebral column and uniform distribution of the hyperbaric Bupivacaine solution, by tilting the table toward the right side.

Our rationale for tilting the table to 20° toward the right side was based on previous experiences and many studies, which clearly indicate the movement of the anesthetic solution to the lower end of the spinal column where it subsequently spreads unilaterally, due to spinal deformity.

In one similar case injected a hypobaric anesthetic solution in the right lateral position and maintained the same position to obtain bilateral surgical anesthesia.

This case demonstrates that Taylor's approach can be successful even in cases of severe lumbosacral deformities. Intrathecal injection of a hyperbaric local anesthetic, along with the optimal position, that is the desired angle, may help achieve symmetrical and adequate motor and sensory blockade in patients with extreme spinal deformities.

## CONCLUSION

Patients with chronic diseases of the spine represent specific challenges to the anesthesiologist. Handling of the airways and the access to the neuroaxis can be difficult. Most anesthesiologists prefer to use general anesthesia in these patients, avoiding the neuroaxis, despite the presence of difficult airways. The degree of spine involvement will determine how difficult the tracheal intubation could be. Special care should be taken to avoid excessive manipulation of the neck, which could cause trauma to the spinal cord.

Our case highlights that in some cases of ankylosing spondylitis one can be successful in using the modified approach to subarachnoid block, the Taylor's approach, which is considered to be technically more difficult than the standard midline approach, which is considered to be technically easier.
